# Unhealthy food consumption and its associated factors among infants and young children in Gondar city, northwest Ethiopia: a community based cross sectional study

**DOI:** 10.1186/s40795-023-00722-z

**Published:** 2023-05-25

**Authors:** Deresegne Fentie Jemere, Mekonnen Sisay Alemayehu, Aysheshim Kassahun Belew

**Affiliations:** 1 Central Gondar Zonal Health Department, Gondar, Ethiopia; 2 Department of Human Nutrition, Institute of Public Health, University of Gondar, Gondar, Ethiopia

**Keywords:** Ethiopia, Gondar city, Infants and young children, Unhealthy food

## Abstract

**Introduction:**

Many low- and middle-income countries are now shifting toward diets that are higher in added sugars, unhealthy fats, salt, and refined carbohydrates. Childhood obesity and chronic diseases have all been linked to unhealthy food consumption. Despite this, the majority of Ethiopian infants and children consume unhealthy food. There is also a scarcity of evidence. Therefore, the objective of this study was to assess the prevalence of unhealthy food consumption and its associated factors among children ages 6–23 months in Gondar City, northwest Ethiopia.

**Methods:**

A community-based cross-sectional study was conducted from June 30 to July 21, 2022, in Gondar city. Multistage sampling was used to select 811 mother-child pairs. Food consumption was measured through a 24-hour recall. Data were entered into EpI Data 3.1 before being exported to STATA 14 for further analysis. A multivariable logistic regression analysis was employed to identify the factors associated with unhealthy food consumption. An adjusted odds ratio (AOR) with a 95% confidence interval was used to show the strength of the association, while a P-value of 0.05 was used to declare the significance of the association.

**Results:**

The percentage of children with unhealthy food consumption was 63.7% (95% CI: 60.4%, 67.2%). Maternal education [AOR = 1.89, 95% CI = 1.05, 3.69], living in an urban residence [AOR = 4.55, 95% CI = 3.61, 7.78], GMP service [AOR = 2.07, 95% CI = 1.48, 3.18], age of the child 18–23 months [AOR = 0.53, 95% CI = 0.34, 0.74], and family size of more than four [AOR = 1.22, 95% CI = 1.07, 2.78] were significantly associated with unhealthy food consumption.

**Conclusion:**

In Gondar City, nearly two thirds of infants and children received unhealthy food. Maternal education, urban residence, GMP service, child age, and family size were all significant predictors of unhealthy food consumption. Thus, improving the uptake of GMP services and family planning services is critical to reducing unhealthy food consumption.

## Introduction

Many low- and middle-income countries (LMICs) are going through a nutritional transition, with diets shifting toward more added sugars, unhealthy fats, salt, and refined carbohydrates [1]. As a result, children in both LMICs and industrialized countries consume more unhealthy foods and sugary drinks [2, 3]. Sugar-sweetened beverages are related to an increased risk of being overweight and obese due to excessive energy intake [4, 5]. Currently, a high intake of unhealthy foods throughout infancy and early childhood has been related to overweight and obesity [6–10].

Globally, 40 (5.9%) million children under the age of five are overweight or obese, with the majority of these children living in low- and middle-income countries [11]. Central Asia (14.9%) and the Middle East and North Africa (11.2%) have the highest rates of overweight [12]. According to the Ethiopia Demographic Health Survey (EDHS), the number of overweight children increased from 1 to 2% between 2016 and 2019 [13, 14]. Overweight and obesity rank as the fifth leading risk factor for mortality, and morbidity is one of the major public health problems, causing 45% of all deaths among children aged 0–59 months [15].

Childhood obesity has consequences for adulthood obesity, which can lead to psychosocial problems as well as cardiovascular risk factors such as high blood pressure, high cholesterol, diabetes, and sleep disordered breathing [16–18]. In addition, low self-esteem increases the likelihood of being bullied, as does poor school attendance and achievement, a low employment rate, and a lower-paying job in adulthood [19, 20]. Adult obesity also costs the world $ 2.0 trillion per year [21].

Reports show that food marketing environments [22], maternal occupation [23], the child’s first appointment at the primary healthcare facility [23], lower income households [23], TV food advertising [24], urban residence [25], the age of the child [25], and being enrolled in cash transfer programs [23] are the factors for increasing the consumption of unhealthy food.

Ethiopia has various initiatives and programs in place to minimize all forms of malnutrition, including overweight and obesity, as part of its national development plan since children under the age of two have been identified as being part of the first key window of opportunity [26]. However, the prevalence of obesity and overweight in Ethiopia ranges from 1.7 to 3.6% [27]. Furthermore, less attention has been paid to the increased accessibility of unhealthy foods, suboptimal feeding practices, and the rising trend in overweight/obesity. Regular monitoring of infant and child feeding practices helps to provide baseline evidence as well as monitor and evaluate current initiatives. However, little is known, including in the research area, regarding the prevalence of unhealthy food consumption among children aged 6 to 23 months in Ethiopia. As a result, the purpose of this study was to determine the prevalence and associated factors of unhealthy food consumption in 6 to 23 month old children in Gondar City, Northwest Ethiopia.

## Methods

### Study design, period and area

A community-based cross-sectional study was conducted from June 30 to July 21, 2022, in Gondar City, Northwest Ethiopia. Gondar City is the capital city of Gondar city administration and the central Gondar zone, which has six sub-cities. It is 727 km from Addis Ababa, the Federal Democratic Republic of Ethiopia’s capital, and 120 km from Bahir Dar, the capital of the Amhara National Regional State. The city has 3 hospitals, 8 health centers, and 14 health posts. The city has a total population of 454,445 people, including 22,945 children under the age of two.

### Source population and study population

All infants and young children aged between 6 and 23 months, with their mothers or caregivers, living in Gondar city were considered the source population, whereas all infants and young children aged between 6 and 23 months, with their mothers or caregivers, in selected Kebeles living in Gondar city were considered the study population.

### Sample size and sampling procedure

Based on the following assumptions, a sample size was calculated by using the single population formula: Because there have been no previous studies, a 95% CI was calculated using 50% of the previous prevalence, a 5% margin of error, and a 10% non-response rate. Finally, a total of 845 samples were obtained with 2% design effects. To identify study participants, a simple random sampling procedure was used. A list of infants and children was found in health posts. First, two sub-cities were selected from the total number of sub-cities in Gondar by using the lottery method. Second, six Kebeles were selected from each of the selected sub-cities by using the lottery method. Third, after having the number of all 6–23-month-old children from the selected kebeles, corresponding to the month of the 6-23-month-old children, samples were allocated proportionally to each kebele. Finally, a total of 845 participants were taken from each kebele through a simple random sampling technique.

### Data collection procedures and tools

The data collection instruments were structured questionnaires that were pretested. The questionnaire was adapted from the World Health Organization (WHO) Infant and Young Child feeding (IYCF) indicators and other literature with some modifications [28–32]. The questionnaire was prepared originally in English and translated from Amharic back to English. It contains different independent variables, like the socio-demographic characteristics of parents, infants, and young children; household-related factors; and maternal health services related factors. The single 24-hour recall method was used to measure infants’ and children’s food consumption. There were four BSc nurse data collectors and one public health nutritionist supervisor assigned.

### Data quality control

Two-day training was given on the contents of the questionnaire, interviewing technique, the purpose of the study, and how the principal investigator approaches and maintains the respondents’ confidentiality. A pre-test was conducted with 5% of the total sample size. The quality of the data was checked every day after data collection, and any errors were corrected before the following data gathering measures. Prior to analysis, the data was cleaned up and cross-checked. The principal investigator and the supervisor closely monitored the process throughout the data collection period and made any necessary corrections.

### Variable measurnemts

To determine each child’s unhealthy food consumption (UFC), the mother was asked to list all foods consumed by the child in the 24 h preceding the survey, such as Juices, soda, coffee or tea with sugar, candies, chocolate, cakes, sweet biscuits, ice cream, potato chips and instant noodle. Children who got at least one food from the above lists were classified as meeting the unhealthy food consumption; otherwise, they were considered to be getting healthy food consumption [32].

A mother with less than two-year-old children who read a newspaper or magazine, listened to the radio, or watched television at least once per week is considered to have satisfactory media exposure; children who did so less than once per week are considered to have unsatisfactory media exposure.

Postnatal care (PNC) is defined as care provided to the mother and her newborn baby immediately following placental birth and for the first 42 days of life [33].

GMP utilization was measured by taking a child’s GMP and having them participate in GMP at least once in the previous three months.

The household wealth index was determined using principal component analysis (PCA) by considering household assets, such as quantity of cereal products, house, livestock, and agricultural land ownership. First, variables were coded between 0 and 1. Then, the variables were entered and analyzed using PCA, and those variables having a communality value of greater than 0.5 were used to produce factor scores. Finally, the factor scores were summed and ranked into poor, medium, and rich wealth index [34].

### Data processing and analysis

Cleaned and coded data was entered into EPI Data Version 3.1 and exported to STATA Version 14 for further analysis. Descriptive and summary statistics were presented in the form of text, tables, and graphs. Both frequency and tables are used to summarize descriptive statistics. Variables with a p-value less than 0.2 in the bivariable analysis were fitted into the multivariable logistic regression analysis. A multivariable logistic regression analysis was employed to identify the factors associated with unhealthy food consumption. An adjusted odds ratio (AOR) with a 95% confidence interval was used to show the strength of the association, while a P-value of 0.05 was used to declare the significance of the association.

## Results

### Socio-demographic characteristics

A total of 811 mother-child pairs with children aged 6–23 months were enrolled in the study, with a response rate of 96%. The mean age of the respondents was 32.33 years, with a standard deviation of 6.24. More than one-third of the mothers, 277 (34.1%), were between the ages of 25 and 30. More than three-quarters of the respondents, 631 (77.8%) and 608 (75%), were married and Orthodox Christians, respectively. More than one third, 283 (34.9%), of the mothers were housewives. Less than a quarter, 182 (22.4%), and 160 (19.7%) of the mothers and fathers, were able to read and write their educational status, respectively. Housewives made up less than half of the 349 mothers (43%) (Table 1).

**Table 1:** Parental level characteristics of children aged 6–23 months in Gondar City, Northwest Ethiopia, 2022

Variables	Frequency	Percentage
**Age of the mother in year**		
18–24	101	10.1
25–30	277	34.1
31–35	191	23.5
≥ 36	261	32.3
**Religion**		
Orthodox	608	75
Muslim	111	13.7
Protestants	60	7.4
Catholic	32	3.9
**Marital status**		
Married	631	77.8
Un married	180	22.2
**Maternal educational status**		
Unable to read and write	116	14.3
Able to read and write	182	22.4
primary school	129	15.9
Secondary school	177	21.8
Diploma and above	207	25.5
**Occupation of the mother**		
House wife	349	43
Merchant	252	31.1
Government	123	15.2
NGO	11	1.4
Daily labor	43	5.3
Student	33	4.1
**Caregiver relation to the child**		
Grandmother	71	8.8
Mother	740	91.2
**Educational status of the Father**		
Unable to read and write	54	6.7
Able to read and write	160	19.7
Primary school	140	17.3
Secondary school	178	21.9
Certificate and Diploma	127	15.7
Degree and above	152	18.7
**Father’s Occupation**		
Merchant	446	55
Government employee	184	22.7
NGO	22	2.7
Farmer	89	11
Daily laborer	53	6.5
Student	17	2.1

### Child, household, and community-related characteristics

More than one-third, 297 (36.6%), of the children were within the age range of 12 to 17 months. The majority of study participants, 685 (84.5%), lived in urban Kebeles. More than half, 485 (59.8%) of respondents, had fewer than or equal to four family members. Most 724 (89.3%) of the participants did not have a garden at home. More than two-thirds 620 (76.40%) of the study participants, received advice on child feeding practices from health professionals. More than two thirds, 624 (76.94%) of the participants, began providing their children with complementary feeding, and 604 (74.5%) of the respondents had satisfactory media exposure. The vast majority of children, 730 (90%), are currently breastfed (Table 2).

**Table 2:** Child, household, and community characteristics of children aged 6–23 months in Gondar City, Northwest, Ethiopia, 2022

Variables	Frequency	Percentage (%)
**Age of the Child**		
6–11 months	249	30.7
12–17 months	297	36.6
18–23 months	265	32.7
**Sex of the child**		
Female	404	49.8
Male	407	50.2
**Birth Order**		
First	284	35
Second to fourth	496	61.2
4th and above	31	3.8
**Number of under five children**		
One	609	75.1
Two	186	22.9
Three and above	16	2.0
**Family Size**		
<=4	485	59.8
> 4	326	40.2
**Residence**		
Urban	685	84.5
Rural	126	15.5
**Household wealth status**		
Poor	271	33.4
Middle	270	33.3
Rich	270	33.3
**Media exposure**		
Unsatisfactory	142	17.5
satisfactory	669	82.5
**Home Gardening**		
No	724	89.7
Yes	87	10.7
**Current breastfeed**		
No	81	10
Yes	730	90
**Complementary feeding**		
No	187	23.1
Yes	624	76.9

### Child, and health care level related characteristics

Nearly two thirds, 494 (60.9%) of the respondents’ mothers, had four and above ANC visits during their last pregnancies. Almost three-quarters 579(71.4%) of the mothers received information on infant feeding during any of their antenatal care (ANC) visits. The majority, 720 (88.8%) of the study participants, had PNC visits during their last pregnancies, and nearly two-thirds of 500 (61.7%) of the children had growth monitoring and promotion (GMP) in the last three months. Regarding the birth place, the majority 773, (95.3%) of children were from health facilities (Table 3**).**

**Table 3:** Child and health care level related factors among 6-23months of child in Gondar City, Northwest Ethiopia, 2022

Variables	Frequency	Percentage (%)
**ANC Visits**		
No	37	4.6
Yes	774	95.4
**Number of ANC Visits**		
None	37	4.6
One to three times	280	34.5
Four and above visits	494	60.9
**Obtained infant feeding Information during ANC**		
No	232	28.6
Yes	579	71.4
**Place of Birth**		
Home	38	4.7
Health facilities	773	95.3
**PNC Visits**		
No	91	11.2
Yes	720	88.8
**Obtained infant feeding Information during PNC**		
No	259	31.4
Yes	552	68.6
**GMP service**		
No	311	38.3
Yes	500	61.7

### Prevalence of unhealthy food consumption

The overall prevalence of unhealthy food consumption among children aged 6–23 months was 63.7% (95% (CI: 60.4, 67.2%).

### Factors associated with unhealthy food consumption

The multivariable logistic regression revealed that educational status of the mother, age of the child; urban residence, family size, and GMP service were all significantly associated with unhealthy food consumption (Table 4).

**Table 4:** Multivariable logistic regression of variables for unhealthy food consumption among children 6-23months of age in Gondar City, Northwest Ethiopia, 2022

Variables	Unhealthy food consumption		COR [95% CI]	AOR [95%CI]
	Yes	No		
**Age of mothers in years**				
18–24	50(61%)	32(39%)	1.32(0.79, 2.27)	0.85(0.45, 1.61)
25–30	172(62.1%)	105(37.9%)	1.26(0.89, 1.80)	1.01(0.65, 1.57)
31–35	119(62.3%)	72(37.7%)	1.25(0.85, 1.85)	0.84(0.53, 1.35)
≥ 36	176(67.4%)	85(32.6%)	1	1
**Maternal education**				
Unable to read &write	56(48.3%)	60(51.7%)	2.89(1.79, 4.65)	1.89(1.05, 3.69)*
Read and write	113(62.1%)	69(37.9%)	1.65(1.07, 2.53)	1.16(0.65, 2.08))
Primary school	81(62.8%)	48(37.2%)	1.60(0.99, 2.56)	1.39(0.76, 2.55)
Secondary school	116(65.5%)	61(34.5%)	1.42(0.92,2.19)	1.37(0.80, 2.37)
Diploma and above	151(72.9%)	56(27.1%)	1	1
**Mother’s Occupation**				
House wife	187(53.6%)	162(46.4%)	1	1
Merchant	178(70.6%)	74(29.4%)	0.48(0.34, 0.68)	0.39(0.29, 0.47))
Government Employee	94(76.4%)	29(23.6%0	0.36(0.22, 0.57)	0.33(0.19, 0.43)
Daily labor	32(74.4%)	11(25.6%)	0.40(0.19, 0.81)	0.35(0.21,0.53)
Students	17(51.5%)	16(45.5%)	1.09(0.53, 2.22)	0.89(0.72, 0.89)
NGO	9(81.8%)	2(18.2%)	0.26(0.06, 1.20)	0.18(0.05, 0.87)
**Father’s education**				
Unable to read &write	25(46.3%)	29(53.7%)	2.51(1.33, 4.74)	0.94(0.35, 2.54)
Read and write	97(60.6%)	63(39.4%)	1.41(0.88, 2.24)	0.52(0.24, 1.12)
Primary school	88(62.9%)	52(37.1%)	1.28(0.79, 2.08)	0.77(0.39, 1.52)
Secondary school	117(65.7%)	61(34.3%)	1.13(0.71, 1.79)	0.85(0.45, 1.87)
Diploma	86(67.3%)	41(32.3%)	1.03(0.62, 1.71)	0.90(0.49, 2.04)
Degree and above	104(68.4%)	48(31.6%)	1	1
**Age of the child**				
6–11 months	151(58.1%)	109(41.9%)	1	
12–17 months	200(63.5%)	115(36.5%)	0.79(0.57,1.12)	0.71(0.39, 1.09)
18–23 months	166(70.3%)	70(29.7%)	0.58(0.40,0.85)	0.53(0.34, 0.74)*
**Family size**				
≤ 4 families	296(61%)	189(39%)	1	1
> 4 families	221(67.8%)	105(32.2%)	1.34(1.21, 1.82)	1.22(1.07, 2.78)*
**Residence**				
Urban	500(72.2%)	193(27.8%)	5.39(3.97, 9.41)	4.55(3.61, 7.78)*
Rural	17(14.4%)	101(85.6%)	1	1
**PNC**				
No	157(57.9%)	114(42.1%)	1.45(1.07, 1.96)	0.96(0.65, 1.43**)**
Yes	360(66.7%)	180(33.3%)	1	1
**GMP Service**				
No	164(52.9%)	147(42.1%)	2.15(1.60, 2.89)	2.07(1.48, 3.18)*
Yes	353(70.6%)	147(29.4%)	1	1
**Wealth index**				
Poor	159(58.7%)	112(41.3%)	1.01(0.72, 1.42)	0.79(0.52, 1.22)
Middle	199(73.7%)	71(26.3%)	0.51(0.36, 0.74)	0.42(0.33, 0.71)
Rich	159(58.9%)	111(41.1%)	1	1
**Media exposure**				
Unsatisfactory	45(31.7%)	97(68.3%)	5.17(3.49, 7.63)	4.07(3.25, 6.96)
Satisfactory	472(70.6%)	197(29.4%)	1	1

*indicate p value less than 0.05

Mothers unable to read and write were 1.89 times [AOR = 1.89, 95%CI = 1.05, 3.69] more likely to consume unhealthy food as compared to those with a diploma and above.

Children aged 18–23 months were 47% less likely [AOR = 0.53; 95% CI = 0.34; 0.74] to consume unhealthy food than those aged 6–11 months.

Children living in families larger than four were 1.22 times more likely to consume unhealthy food than those living in families smaller than or equal to four [AOR = 1.22, 95% CI = 1.07, 2.78].

Moreover, mothers who lived in urban areas were 4.55 times more likely [AOR = 4.55, 95% CI = 3.61, 7.78] to provide unhealthy food as compared to mothers who lived in rural areas.

Mothers who had not received GMP services were 2.07 [AOR = 2.07, 95% CI = 1.48, 3.18] times more likely to provide unhealthy foods as compared with mothers who had received GMP services.

## Discussion

Unhealthy food consumption among children aged 6–23 months in Gondar city was 63.7%. This finding is consistent with a study done in Iran (66.8%) [35]. Whereas, this report is lower than a study done in Nepal (74.1%) [36]. The possible reason might be due to different sociodemogrphic characteristics, cultural variation, and study design. However, this finding is higher than in Tanzania (23.10%) [37], Dakar (58.7%) [29], and Peru (19.30%) [38]. One possible explanation is that the Peru study was conducted during the COVID-19 pandemic, which limited market access to unhealthy foods [39].

Mothers unable to read and write were 1.89 times more likely to consume unhealthy food as compared to those with a diploma and above. The possible explanation might be that women who are unable to read or write are unconcerned about the short- and long-term consequences of unhealthy food consumption [40]. In addition, mothers who are unable to read or write will have poor knowledge of nutrients, limited access to high-quality food sources, lower incomes, and are less likely to buy recommended health foods, preferring instead to buy sugary beverages or sweet baked foods [41, 42].

Children living in families larger than four were 1.22 times more likely to consume unhealthy food than those living in families smaller than or equal to four. This could be due to the fact that mothers with larger families take longer to prepare foods and, as a result, purchase unhealthy items for their family. Furthermore, intra-household food distribution may boost the desire to consume unhealthy foods [43]; unhealthy foods are less expensive and easier to prepare than healthy foods [28].

Moreover, mothers who lived in urban areas were 4.55 times more likely to provide unhealthy food as compared to mothers who lived in rural areas. The finding is supported by the global food policy report [44]. The possible reason might be due to the fact that mothers living in urban residences may have the chance to access and purchase different kinds of unhealthy food from small shops, supermarkets, and street food vendors to provide unhealthy food for their children. Mothers who are living residences may be affected by food insecurity [45], shifting to purchasing less costly food from the market [46]. The other possible reason might be the urban poor population, for whom the most easily available and affordable diets are often unhealthy foods [47].

Children aged 18–23 months were 47% less likely to consume unhealthy food than those aged 6–11 months. Our finding is contradicted by studies done in Africa, Asia [29], and Nepal [48]. Possible reasons include child preference and a strong demand for sweet and convenient foods to begin complementary feeding at a young age. In addition, 18- to 23-month-old children have a higher probability of eating a family diet, which decreases unhealthy food consumption [49].

Mothers who hadn’t had GMP visits were 2.07 times more likely to provide unhealthy foods as compared to mothers who had GMP visits. The possible reason might be due to the fact that GMP focuses on empowering mothers to know about and become competent in appropriate child care and feeding practices through individual and group child feeding practices [50]. GMP is one of the techniques used to develop mothers’ and caregivers’ awareness, knowledge, and skill in preparing a diverse diet at home through community conversion and porridge preparation rather than providing unhealthy food for their children [51].

### Strength and limitation of the study

The strength of this study was that it was the first of its kind in the study area targeting unhealthy food consumption among children aged 6–23 months. As a limitation, the data collection was done based on past feeding practices, which might be socially desirable. Since the data collection was done in a single 24-hour recall, the finding did not indicate the usual dietary habit of an individual child.

## Conclusion

In Gondar City, nearly two thirds of infants and children received unhealthy food. Maternal education, urban residence, GMP service, child age, and family size were all significant predictors of unhealthy food consumption. Thus, improving the uptake of GMP services and family planning services is critical to reducing unhealthy food consumption.

## Data Availability

Data will be available upon request from the corresponding authors.

## Abbreviations

AOR: Adjusted Odds Ratio
ANC: Antenatal Care
BSC: Bachelors of Science
CI: Confidence Interval
COR: Crud Odds Ratio
EDHS: Ethiopian Demographic and Health Survey
GMP: Growth Monitoring and promotion
LMICs: Low- and middle-income countries
IYCF: Infant and young child feeding
PCA: Principal component analysis
PNC: Postnatal care
UFC: Unhealthy Food Consumption
WHO: World Health Organization.
